# 1-(2-Pyrid­yl)-*N*,*N*′-dipyrimidin-2-ylmethane­diamine

**DOI:** 10.1107/S1600536808032583

**Published:** 2008-10-15

**Authors:** Masoumeh Tabatabaee, Fatemeh Hakimi, Mina Roshani, Mohammad Mirjalili, Hamid Reza Kavasi

**Affiliations:** a Department of Chemistry, Islamic Azad University, Yazd Branch, Yazd, Iran; b Department of Chemistry, Islamic Azad University, Mashhad Branch, Mashhad, Iran; c Department of Textile Engineering, Yazd, Iran; d Department of Chemistry, Shahid Beheshti University, Tehran, Iran

## Abstract

In the title compound, C_14_H_13_N_7_, inter­molecular N—H⋯N and C—H⋯N hydrogen bonds link the mol­ecules into infinite one-dimensional chains along (100). A C—H⋯π inter­action also occurs in the crystal.

## Related literature

For the biological activity of pyrimidine derivatives, see: Onal & Altral (1999 ▶); Ponticelli & Spanu (1999 ▶). For their uses in coordination chemistry, see: Prince *et al.* (2003 ▶); Lee *et al.* (2003 ▶); Masaki *et al.* (2002 ▶). For studies of the reactions of heterocyclic amines with aromatic aldehyde to prepare new ligands, see: Tabatabaee *et al.* (2006 ▶, 2007*a* ▶,*b* ▶, 2008 ▶).
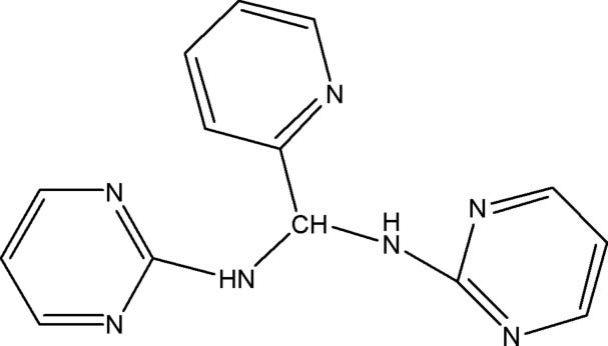


## Experimental

### 

#### Crystal data

C_14_H_13_N_7_
*M* *_r_* = 279.31
Monoclinic, 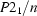

*a* = 9.5781 (19) Å
*b* = 9.3543 (16) Å
*c* = 15.975 (4) Å
β = 97.521 (17)°
*V* = 1419.0 (5) Å^3^
*Z* = 4
Mo *K*α radiation
μ = 0.09 mm^−1^
*T* = 296 K
0.45 × 0.20 × 0.05 mm

#### Data collection

Stoe IPDS-II diffractometer
Absorption correction: numerical [shape of crystal determined optically; *X-SHAPE* and *X-RED32* (Stoe & Cie, 2005 ▶)]*T*_min_ = 0.970, *T*_max_ = 0.998
12281 measured reflections
2997 independent reflections
2443 reflections with *I* > 2σ(*I*)
*R*_int_ = 0.032

#### Refinement

*R*[*F* ^2^ > 2σ(*F* ^2^)] = 0.058
*wR*(*F* ^2^) = 0.140
*S* = 1.12
2997 reflections
242 parameters
All H-atom parameters refined
Δρ_max_ = 0.15 e Å^−3^
Δρ_min_ = −0.16 e Å^−3^

### 

Data collection: *X-AREA* (Stoe & Cie, 2005 ▶); cell refinement: *X-AREA*; data reduction: *X-RED32* (Stoe & Cie, 2005 ▶); program(s) used to solve structure: *SHELXS97* (Sheldrick, 2008 ▶); program(s) used to refine structure: *SHELXL97* (Sheldrick, 2008 ▶); molecular graphics: *ORTEP-3 for Windows* (Farrugia, 1997 ▶); software used to prepare material for publication: *WinGX* (Farrugia, 1999 ▶).

## Supplementary Material

Crystal structure: contains datablocks I, global. DOI: 10.1107/S1600536808032583/bq2098sup1.cif

Structure factors: contains datablocks I. DOI: 10.1107/S1600536808032583/bq2098Isup2.hkl

Additional supplementary materials:  crystallographic information; 3D view; checkCIF report

## Figures and Tables

**Table 1:** Hydrogen-bond geometry (Å, °) *Cg*1 is the centroid of the N6/N7/C11–C14 ring.

*D*—H⋯*A*	*D*—H	H⋯*A*	*D*⋯*A*	*D*—H⋯*A*
N2—H2*B*⋯N4^i^	0.885 (19)	2.181 (19)	3.064 (2)	175.3 (19)
N5—H5*B*⋯N7^ii^	0.787 (19)	2.259 (19)	3.042 (3)	174 (2)
C12—H12⋯N3^iii^	1.00 (3)	2.57 (2)	3.304 (3)	130.4 (15)
C9—H9⋯*Cg*1^iv^	0.99 (3)	2.62 (3)	3.532 (3)	154 (3)

Symmetry codes: (i) 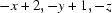; (ii) 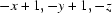; (iii) 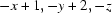; (iv) 
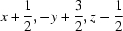
.

**Fig. 1.**
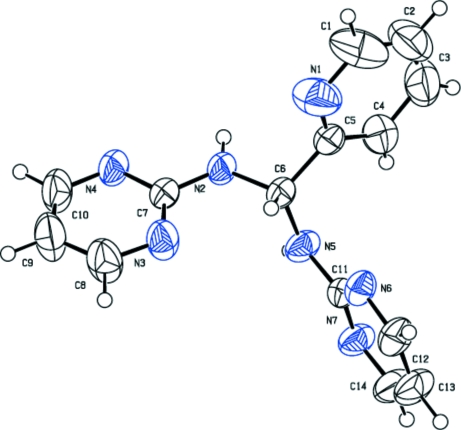
The molecular structure of (I). Displacement ellipsoids are drawn at the 50% probability level.

**Fig. 2.**
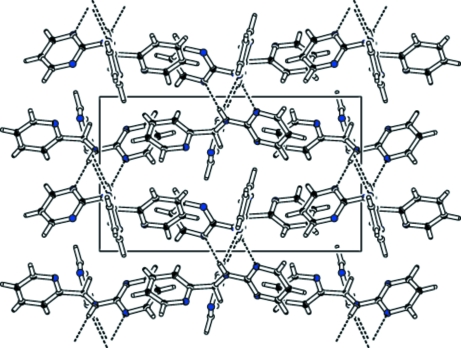
Packing diagram of (I), molecules are linked into infinite one dimensional chains by hydrogen-bond interactions (dashed lines).

**Fig. 3.**
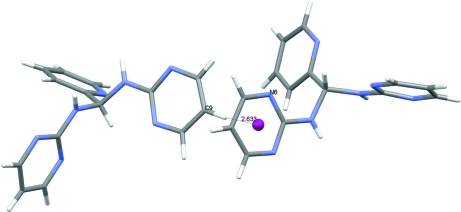
Intermolecular C—H···\p interaction between one aromatic pyrimidine ring and adjacent molecule.

## supplementary crystallographic information

### Comment

Pyrimidines derivatives possess remarkable biological activity and have been widely used in medicinal and industrial applications (Onal & Altral, 1999; Ponticelli *et al.* 1999). Moreover amino pyrimidine derivatives find use in coordination chemistry (Prince *et al.* 2003; Masaki *et al.* 2002; Lee *et al.* 2003). In continuation of our recent work on the reactions of heterocyclic amines with aromatic aldehyde to prepare of new ligands (Tabatabaee *et al.* 2006; Tabatabaee *et al.* 2007a,b; Tabatabaee *et al.* 2008) in this communication, we report, our results on the reaction of 2-aminopyrimidine and 2-pyridinecarbaldehyde. The title compound, C_14_H_13_N_7_, (I), is a new aminoacetal compound obtained from condensation of 2-aminopyrimidine with 2-pyridinecarbaldehyde. The crystal structure of (I) (Fig. 1) shows that one molecule of 2-pyridinecarbaldehyde was reacted with two molecules of 2-aminopyrimidine to form (I). Bond lengths and angles are unexceptional and the molecular structure is stabilized by some intermolecular N—H···N and C—H···N hydrogen-bonds (Table I). In the crystal packing (Fig. 2), molecules are linked into infinite one dimensional chains by hydrogen-bond interactions. A considerable feature of the compound (I) is the presence of C—H···π stacking interactions between C—H groups from one molecule and aromatic pyrimidine ring of adjacent molecule.The C—H···π distance is 2.62 (3) Å for C9—H9···*Cg1* (Cg1 is the center of pyrimidine ring), with the angle of 154 (3)° (Fig.3).

### Experimental

A solution of 2-aminopyrimidine (0.94 g, 1 mmol) in EtOH (15 ml) was treated with 2-pyridinecarbaldehyde (0.107 g, 1 mmol) and the resulting mixture was acidified with 37% hydrochloric acid (0.2 ml). The reaction mixture was refluxed for 8 h. The solid residue was filtered and the filtrate was kept at 293 K. Colorless crystals of the title compound were obtained after a few days (yield 83%).

### Refinement

All of the H atoms were located in a difference synthesis and refined isotropically [aromatic C—H = 0.82 (2)–1.07 (3) Å, tertiary C—H = 0.97 (2) Å and N—H = 0.79 (2)–0.89 (2) Å] and refined isotropically.

### Crystal data

**Table:**

C_14_H_13_N_7_	*F*(000) = 584
*M**_r_* = 279.31	*D*_x_ = 1.307 Mg m^−^^3^
Monoclinic, *P*2_1_/*n*	Mo *K*α radiation, λ = 0.71073 Å
Hall symbol: -P 2yn	Cell parameters from 2000 reflections
*a* = 9.5781 (19) Å	θ = 2.4–26.8°
*b* = 9.3543 (16) Å	µ = 0.09 mm^−^^1^
*c* = 15.975 (4) Å	*T* = 296 K
β = 97.521 (17)°	Plate, colorless
*V* = 1419.0 (5) Å^3^	0.45 × 0.20 × 0.05 mm
*Z* = 4	

### Data collection

**Table:**

Stoe IPDS-II diffractometer	2997 independent reflections
Radiation source: fine-focus sealed tube	2443 reflections with *I* > 2σ(*I*)
graphite	*R*_int_ = 0.032
rotation method scans	θ_max_ = 26.8°, θ_min_ = 2.4°
Absorption correction: numerical shape of crystal determined optically (Program? reference?)	*h* = −12→12
*T*_min_ = 0.970, *T*_max_ = 0.998	*k* = −11→11
12281 measured reflections	*l* = −20→17

### Refinement

**Table:**

Refinement on *F*^2^	Primary atom site location: structure-invariant direct methods
Least-squares matrix: full	Secondary atom site location: difference Fourier map
*R*[*F*^2^ > 2σ(*F*^2^)] = 0.058	Hydrogen site location: inferred from neighbouring sites
*wR*(*F*^2^) = 0.140	All H-atom parameters refined
*S* = 1.12	*w* = 1/[σ^2^(*F*_o_^2^) + (0.0555*P*)^2^ + 0.3988*P*] where *P* = (*F*_o_^2^ + 2*F*_c_^2^)/3
2997 reflections	(Δ/σ)_max_ = 0.002
242 parameters	Δρ_max_ = 0.15 e Å^−^^3^
0 restraints	Δρ_min_ = −0.16 e Å^−^^3^

### Special details

**Table:**

Geometry. All e.s.d.'s (except the e.s.d. in the dihedral angle between two l.s. planes) are estimated using the full covariance matrix. The cell e.s.d.'s are taken into account individually in the estimation of e.s.d.'s in distances, angles and torsion angles; correlations between e.s.d.'s in cell parameters are only used when they are defined by crystal symmetry. An approximate (isotropic) treatment of cell e.s.d.'s is used for estimating e.s.d.'s involving l.s. planes.
Refinement. Refinement of *F*^2^ against ALL reflections. The weighted *R*-factor *wR* and goodness of fit *S* are based on *F*^2^, conventional *R*-factors *R* are based on *F*, with *F* set to zero for negative *F*^2^. The threshold expression of *F*^2^ > σ(*F*^2^) is used only for calculating *R*-factors(gt) *etc*. and is not relevant to the choice of reflections for refinement. *R*-factors based on *F*^2^ are statistically about twice as large as those based on *F*, and *R*- factors based on ALL data will be even larger.

### Fractional atomic coordinates and isotropic or equivalent isotropic displacement parameters (Å^2^)

**Table:**

	*x*	*y*	*z*	*U*_iso_*/*U*_eq_	
C1	0.9822 (4)	0.8397 (4)	0.2536 (3)	0.1170 (12)	
H1	1.065 (4)	0.909 (5)	0.265 (3)	0.172 (17)*	
C2	0.9364 (4)	0.7672 (5)	0.3161 (2)	0.1087 (12)	
H2	0.973 (4)	0.786 (4)	0.372 (3)	0.149 (14)*	
C3	0.8303 (4)	0.6738 (5)	0.2982 (2)	0.1054 (12)	
H3	0.783 (4)	0.619 (4)	0.328 (3)	0.141 (14)*	
C4	0.7696 (3)	0.6561 (3)	0.21369 (16)	0.0746 (7)	
H4	0.702 (3)	0.604 (3)	0.2000 (16)	0.074 (8)*	
C5	0.82060 (18)	0.7345 (2)	0.15391 (11)	0.0488 (4)	
C6	0.76338 (17)	0.7253 (2)	0.06118 (11)	0.0458 (4)	
H6	0.7549 (19)	0.821 (2)	0.0380 (12)	0.048 (5)*	
C7	0.87637 (17)	0.6815 (2)	−0.06540 (11)	0.0464 (4)	
C8	0.8208 (3)	0.8159 (3)	−0.18238 (15)	0.0816 (8)	
H8	0.753 (3)	0.896 (3)	−0.2127 (17)	0.098 (8)*	
C9	0.9149 (3)	0.7437 (3)	−0.22277 (16)	0.0859 (8)	
H9	0.928 (3)	0.768 (3)	−0.2817 (19)	0.106 (9)*	
C10	0.9855 (3)	0.6351 (3)	−0.17964 (14)	0.0745 (7)	
H10	1.054 (3)	0.577 (3)	−0.2047 (16)	0.086 (7)*	
C11	0.50462 (17)	0.72192 (19)	0.05133 (10)	0.0441 (4)	
C12	0.3865 (2)	0.9233 (2)	0.07540 (14)	0.0599 (5)	
H12	0.394 (2)	1.026 (3)	0.0921 (14)	0.074 (7)*	
C13	0.2605 (2)	0.8541 (3)	0.05806 (16)	0.0698 (6)	
H13	0.170 (3)	0.897 (3)	0.0602 (16)	0.091 (8)*	
C14	0.2664 (2)	0.7119 (3)	0.03806 (16)	0.0681 (6)	
H14	0.180 (3)	0.652 (3)	0.0280 (15)	0.078 (7)*	
N1	0.9275 (2)	0.8262 (3)	0.17169 (15)	0.0913 (7)	
N2	0.86343 (15)	0.65215 (18)	0.01597 (9)	0.0504 (4)	
H2B	0.915 (2)	0.583 (2)	0.0422 (13)	0.058 (6)*	
N3	0.7991 (2)	0.7869 (2)	−0.10338 (11)	0.0681 (5)	
N4	0.96897 (16)	0.59970 (18)	−0.10065 (10)	0.0565 (4)	
N5	0.62782 (15)	0.65417 (19)	0.04528 (11)	0.0523 (4)	
H5B	0.623 (2)	0.575 (2)	0.0285 (13)	0.055 (6)*	
N6	0.51088 (15)	0.85980 (16)	0.07449 (10)	0.0505 (4)	
N7	0.38702 (15)	0.64257 (18)	0.03335 (11)	0.0569 (4)	

### Atomic displacement parameters (Å^2^)

**Table:**

	*U*^11^	*U*^22^	*U*^33^	*U*^12^	*U*^13^	*U*^23^
C1	0.116 (3)	0.121 (3)	0.100 (3)	−0.013 (2)	−0.041 (2)	−0.015 (2)
C2	0.116 (3)	0.145 (3)	0.0593 (18)	0.041 (2)	−0.0115 (17)	−0.023 (2)
C3	0.108 (2)	0.148 (3)	0.0659 (18)	0.040 (2)	0.0316 (17)	0.026 (2)
C4	0.0699 (15)	0.0954 (18)	0.0600 (14)	0.0035 (14)	0.0142 (11)	0.0109 (12)
C5	0.0422 (9)	0.0545 (10)	0.0499 (10)	0.0134 (8)	0.0070 (7)	−0.0050 (8)
C6	0.0405 (8)	0.0518 (10)	0.0466 (9)	0.0107 (7)	0.0112 (7)	−0.0019 (8)
C7	0.0407 (8)	0.0538 (10)	0.0455 (9)	0.0083 (7)	0.0090 (7)	−0.0013 (7)
C8	0.1015 (18)	0.0871 (17)	0.0587 (13)	0.0359 (15)	0.0206 (12)	0.0189 (12)
C9	0.112 (2)	0.0959 (19)	0.0560 (13)	0.0323 (16)	0.0355 (13)	0.0144 (12)
C10	0.0840 (15)	0.0874 (16)	0.0574 (13)	0.0288 (13)	0.0289 (11)	−0.0031 (11)
C11	0.0417 (9)	0.0548 (10)	0.0366 (8)	0.0108 (7)	0.0072 (6)	−0.0032 (7)
C12	0.0564 (11)	0.0512 (11)	0.0751 (14)	0.0147 (9)	0.0204 (10)	−0.0017 (10)
C13	0.0472 (11)	0.0715 (14)	0.0916 (16)	0.0217 (10)	0.0126 (10)	−0.0089 (12)
C14	0.0410 (10)	0.0758 (15)	0.0865 (16)	0.0063 (10)	0.0041 (9)	−0.0215 (12)
N1	0.0819 (14)	0.0997 (16)	0.0858 (15)	−0.0169 (13)	−0.0136 (11)	0.0007 (12)
N2	0.0471 (8)	0.0599 (10)	0.0458 (8)	0.0211 (7)	0.0127 (6)	0.0045 (7)
N3	0.0792 (12)	0.0745 (12)	0.0535 (10)	0.0336 (10)	0.0197 (8)	0.0095 (8)
N4	0.0549 (9)	0.0641 (10)	0.0533 (9)	0.0174 (8)	0.0173 (7)	−0.0023 (7)
N5	0.0394 (8)	0.0528 (10)	0.0648 (10)	0.0093 (7)	0.0066 (6)	−0.0165 (8)
N6	0.0480 (8)	0.0486 (8)	0.0575 (9)	0.0088 (7)	0.0162 (7)	−0.0010 (7)
N7	0.0406 (8)	0.0603 (10)	0.0690 (11)	0.0080 (7)	0.0041 (7)	−0.0189 (8)

### Geometric parameters (Å, °)

**Table:**

C1—C2	1.327 (6)	C8—C9	1.355 (3)
C1—N1	1.350 (4)	C8—H8	1.07 (3)
C1—H1	1.03 (4)	C9—C10	1.357 (4)
C2—C3	1.343 (6)	C9—H9	0.99 (3)
C2—H2	0.93 (4)	C10—N4	1.334 (3)
C3—C4	1.408 (4)	C10—H10	0.98 (3)
C3—H3	0.87 (4)	C11—N6	1.341 (2)
C4—C5	1.346 (3)	C11—N7	1.348 (2)
C4—H4	0.82 (2)	C11—N5	1.354 (2)
C5—N1	1.338 (3)	C12—N6	1.333 (2)
C5—C6	1.513 (3)	C12—C13	1.366 (3)
C6—N2	1.446 (2)	C12—H12	1.00 (2)
C6—N5	1.452 (2)	C13—C14	1.371 (3)
C6—H6	0.97 (2)	C13—H13	0.96 (3)
C7—N3	1.331 (2)	C14—N7	1.335 (2)
C7—N4	1.349 (2)	C14—H14	1.00 (2)
C7—N2	1.350 (2)	N2—H2B	0.89 (2)
C8—N3	1.334 (3)	N5—H5B	0.79 (2)
			
C2—C1—N1	123.8 (4)	C8—C9—H9	120.8 (17)
C2—C1—H1	121 (2)	C10—C9—H9	122.4 (17)
N1—C1—H1	115 (2)	N4—C10—C9	123.6 (2)
C1—C2—C3	119.2 (3)	N4—C10—H10	114.7 (15)
C1—C2—H2	120 (3)	C9—C10—H10	121.8 (15)
C3—C2—H2	120 (2)	N6—C11—N7	126.46 (15)
C2—C3—C4	119.1 (3)	N6—C11—N5	117.52 (16)
C2—C3—H3	135 (3)	N7—C11—N5	116.02 (16)
C4—C3—H3	106 (3)	N6—C12—C13	123.62 (19)
C5—C4—C3	118.3 (3)	N6—C12—H12	113.6 (13)
C5—C4—H4	118.9 (18)	C13—C12—H12	122.7 (13)
C3—C4—H4	122.7 (18)	C12—C13—C14	116.41 (18)
N1—C5—C4	122.5 (2)	C12—C13—H13	124.8 (16)
N1—C5—C6	114.40 (18)	C14—C13—H13	118.7 (16)
C4—C5—C6	123.1 (2)	N7—C14—C13	123.1 (2)
N2—C6—N5	109.37 (15)	N7—C14—H14	115.2 (14)
N2—C6—C5	109.70 (14)	C13—C14—H14	121.6 (14)
N5—C6—C5	113.47 (15)	C5—N1—C1	117.0 (3)
N2—C6—H6	105.9 (11)	C7—N2—C6	122.37 (15)
N5—C6—H6	109.2 (11)	C7—N2—H2B	119.2 (13)
C5—C6—H6	108.9 (11)	C6—N2—H2B	118.4 (13)
N3—C7—N4	125.93 (16)	C7—N3—C8	115.86 (17)
N3—C7—N2	118.29 (15)	C10—N4—C7	114.85 (17)
N4—C7—N2	115.78 (16)	C11—N5—C6	122.74 (16)
N3—C8—C9	123.0 (2)	C11—N5—H5B	117.0 (15)
N3—C8—H8	114.3 (15)	C6—N5—H5B	120.0 (15)
C9—C8—H8	122.5 (15)	C12—N6—C11	115.09 (17)
C8—C9—C10	116.7 (2)	C14—N7—C11	115.17 (17)
			
N1—C1—C2—C3	0.4 (6)	N5—C6—N2—C7	85.7 (2)
C1—C2—C3—C4	−0.5 (5)	C5—C6—N2—C7	−149.27 (18)
C2—C3—C4—C5	−0.3 (5)	N4—C7—N3—C8	−2.7 (3)
C3—C4—C5—N1	1.1 (4)	N2—C7—N3—C8	176.9 (2)
C3—C4—C5—C6	−179.6 (2)	C9—C8—N3—C7	0.2 (4)
N1—C5—C6—N2	71.8 (2)	C9—C10—N4—C7	−0.6 (4)
C4—C5—C6—N2	−107.6 (2)	N3—C7—N4—C10	2.9 (3)
N1—C5—C6—N5	−165.58 (18)	N2—C7—N4—C10	−176.74 (19)
C4—C5—C6—N5	15.0 (3)	N6—C11—N5—C6	−1.2 (3)
N3—C8—C9—C10	1.8 (5)	N7—C11—N5—C6	178.94 (16)
C8—C9—C10—N4	−1.6 (5)	N2—C6—N5—C11	−154.94 (16)
N6—C12—C13—C14	−0.7 (4)	C5—C6—N5—C11	82.2 (2)
C12—C13—C14—N7	−1.5 (4)	C13—C12—N6—C11	2.9 (3)
C4—C5—N1—C1	−1.1 (4)	N7—C11—N6—C12	−3.3 (3)
C6—C5—N1—C1	179.5 (3)	N5—C11—N6—C12	176.80 (17)
C2—C1—N1—C5	0.4 (5)	C13—C14—N7—C11	1.2 (3)
N3—C7—N2—C6	3.6 (3)	N6—C11—N7—C14	1.4 (3)
N4—C7—N2—C6	−176.73 (17)	N5—C11—N7—C14	−178.76 (18)

### Hydrogen-bond geometry (Å, °)

**Table:**

*D*—H···*A*	*D*—H	H···*A*	*D*···*A*	*D*—H···*A*
N2—H2B···N4^i^	0.885 (19)	2.181 (19)	3.064 (2)	175.3 (19)
N5—H5B···N7^ii^	0.787 (19)	2.259 (19)	3.042 (3)	174 (2)
C4—H4···N5	0.82 (3)	2.53 (3)	2.851 (3)	105 (2)
C6—H6···N3	0.968 (19)	2.373 (19)	2.756 (3)	102.9 (13)
C12—H12···N3^iii^	1.00 (3)	2.57 (2)	3.304 (3)	130.4 (15)
C9—H9···Cg1^iv^	0.99 (3)	2.62 (3)	3.532 (3)	154 (3)

Symmetry codes: (i) −*x*+2, −*y*+1, −*z*; (ii) −*x*+1, −*y*+1, −*z*; (iii) −*x*+1, −*y*+2, −*z*; (iv) *x*+1/2, −*y*+3/2, *z*−1/2.
